# Stem cell factor acts locally in the bone marrow to promote stem and progenitor cell maintenance

**DOI:** 10.64898/2026.09.28.755094

**Published:** 2026-09-29

**Authors:** Maria Angelica Freitas-Cortez, Stefano Comazzetto, Xiang Gao, Bo Shen, Lei Ding, Zhiyu Zhao, Sean J. Morrison

**Affiliations:** 1 Children’s Research Institute and the Department of Pediatrics, University of Texas Southwestern Medical Center, Dallas, TX 75390, USA; 2 Centre for Regenerative Medicine, Institute for Regeneration and Repair, University of Edinburgh, Edinburgh, EH16 4UU, United Kingdom; 3 National Institute of Biological Sciences, Beijing 102206, China; Tsinghua Institute of Multidisciplinary Biomedical Research, Tsinghua University, Beijing 100084, China; 4 Columbia Stem Cell Initiative, Department of Rehabilitation and Regenerative Medicine, Department of Microbiology and Immunology, Columbia University Irving Medical Center, New York, NY 10032, USA.; 5 Howard Hughes Medical Institute, University of Texas Southwestern Medical Center, Dallas, TX 75390, USA

**Keywords:** hematopoietic stem cell, niche, endothelial cells, Leptin Receptor^+^ cells, hematopoiesis, restricted hematopoietic progenitors

## Abstract

Stem cell factor (SCF) from Leptin Receptor-expressing (LepR^+^) mesenchymal stromal cells and endothelial cells is required for hematopoietic stem and progenitor cell maintenance in bone marrow. Consistent with this, *Scf* is highly expressed by LepR^+^ cells and at lower levels by endothelial cells in bone marrow. However, endothelial cells outside the bone marrow produce circulating SCF, raising the question of whether the SCF that promotes stem and progenitor cell maintenance is produced inside or outside bone marrow. We found that *Scf* deletion in spleen and liver endothelial cells reduces SCF levels in the blood without affecting stem or progenitor numbers in the bone marrow. Moreover, deletion of *Scf* from mesenchymal cells in the long bones, but not vertebrae, of *Prx1^Cre^*; *Scf^fl/fl^* mice depletes stem and progenitor cells in long bones but not vertebrae. SCF thus acts locally within the bones in which it is made to promote stem and progenitor cell maintenance.

## INTRODUCTION

Adult hematopoiesis is sustained by hematopoietic stem cells (HSCs), multipotent progenitors, and restricted progenitors that reside in the bone marrow. Stem cell factor (SCF, also known as Kit ligand) is critical for the maintenance of HSCs and restricted hematopoietic progenitors^1,2^. *Scf* is highly expressed by perivascular Leptin receptor-expressing (LepR^+^) mesenchymal stromal cells and at lower levels by endothelial cells in the bone marrow^1,3–6^. HSCs and at least some restricted hematopoietic progenitors reside adjacent to these perivascular LepR^+^ cells^1,3,7^. SCF produced by endothelial cells is selectively required for HSC maintenance^1,3^ while SCF produced by LepR^+^ cells is broadly required for the maintenance of HSCs and restricted hematopoietic progenitors in the bone marrow^3,8^.

SCF is also produced by endothelial cells and other cells outside of the bone marrow^9–11^. This raises the question of whether the SCF that promotes HSC and progenitor maintenance in the bone marrow is produced locally within the bone marrow or outside of the bone marrow. There are membrane-bound and soluble forms of SCF^12^. Membrane-bound SCF may be important for stem and progenitor cell maintenance as HSCs are depleted in *Sl/Sl^d^* mutant mice^13^, which lack membrane-bound SCF but still express soluble SCF^14^; however, the levels of soluble SCF are greatly reduced in these mice^15^ and a recent study found that soluble SCF is more important than membrane bound SCF for HSC maintenance^16^. This emphasizes the need to test if SCF in the blood could have systemic effects on HSC maintenance^16^. Mice with a mixture of wild-type and *Sl/Sl^d^* stromal cells only exhibit normal hematopoiesis in the immediate vicinity of wild-type cells, suggesting that SCF acts locally in the niche^17^. Systemic deletion of membrane-bound, but not soluble, SCF from endothelial cells reduced circulating SCF levels in the blood without having any effect on HSC frequency in the bone marrow^16^. While this observation suggests that HSCs in normal bone marrow do not depend upon SCF from the blood, bones transplanted from fetal *Sl/Sl* mice (which completely lack SCF) into wild-type mice had normal HSC frequency and reduced erythroid progenitor frequency in the marrow of the transplanted bones three weeks after transplantation^16^. This suggests HSCs can be sustained, at least transiently, by soluble SCF produced outside the bone marrow when no SCF is made inside the bone marrow. It is unclear if SCF from the blood contributes to HSC maintenance in the bone marrow under physiological conditions when SCF is synthesized in the bone marrow.

## STUDY DESIGN

To address this question, we deleted *Scf* from endothelial cells in the spleen and liver using *Lyve1^Cre^* (ref^18^) or *Vav1^Cre^* (ref^19^), then examined the effects on SCF levels in the blood and bone marrow as well as HSC and progenitor numbers in the bone marrow. We also deleted *Scf* from mesenchymal cells using *Prx1^Cre^* (ref^20^), which recombines in the long bones but not in vertebrae, then examined the effects on SCF levels in the blood, long bones, and vertebrae as well as HSC and progenitor numbers in the long bones, and vertebrae. Detailed methods are provided in the supplemental Methods.

## RESULTS AND DISCUSSION

The *Vav1^Cre^* allele we used recombines in endothelial cells in the spleen and liver but not in endothelial cells in bone marrow^9^. Spleen and liver endothelial cells express *Scf ^9,22^*. Vav1-cre also recombines in hematopoietic cells, which express little or no *Scf*^1^. Prior studies found that *Vav1^Cre^; Scf^fl/fl^* and littermate control mice did not significantly differ in terms of HSC or restricted progenitor numbers in the bone marrow^1,9^, suggesting that neither hematopoietic cells nor endothelial cells outside of the bone marrow were functionally important sources of SCF for HSC or progenitor maintenance in the bone marrow. We independently tested this, first confirming by qRT-PCR that *Scf* transcript levels were significantly reduced in endothelial cells from the spleen, but not the bone marrow, of *Vav1^Cre^; Scf^fl/fl^* as compared to *Scf^fl/fl^* controls (Supplemental Figure 2A and 2B)^9^. By ELISA analysis, SCF levels were 40% lower in blood serum from *Vav1^Cre^; Scf^fl/fl^* mice as compared to *Scf^fl/fl^* controls (Figure 1A), but SCF levels in bone marrow fluid from femurs and tibias did not significantly differ between *Vav1^Cre^; Scf^fl/fl^* and control mice (Figure 1B). Even modest reductions in SCF levels significantly reduce HSC and progenitor numbers in the bone marrow^1,23^. However, bone marrow cells from *Vav1^Cre^; Scf ^fl/−^* and littermate control mice gave similar levels of donor cell reconstitution upon competitive transplantation into irradiated recipients (Supplemental Figure 2C). These observations, and published data^1,9^, suggest that HSCs and progenitors in the bone marrow do not depend upon SCF from the blood.

We independently assessed this using *Lyve1^Cre^*, which recombines in fetal endothelial cells^24^ and lymphatic endothelial cells^18^. In 3-month-old *Lyve1^Cre^; tdTomato* reporter mice we observed recombination (Tomato^+^) in 11 ± 4.8% of bone marrow endothelial cells, 83 ± 4.0% of spleen endothelial cells, and 88 ± 1.4% of liver endothelial cells (Supplemental Figure 3A). Consistent with this, *Scf* transcript levels were significantly reduced in endothelial cells from the spleen, but not bone marrow, of adult *Lyve1^Cre^;Scf^fl/fl^* mice as compared to *Scf^fl/fl^* controls (Supplemental Figure 3B and 3C). Three month-old *Lyve1^Cre^; Scf^fl/fl^* mice exhibited approximately 50% lower SCF levels in blood plasma (Figure 1C), but not bone marrow fluid, relative to *Scf^fl/fl^* controls (Figure 1D).

Three month-old *Lyve1^Cre^; Scf^fl/fl^* mice and *Scf^fl/fl^* controls did not significantly differ in terms of blood cell counts (Supplemental Figure 3D–O), spleen cellularity (Supplemental Figure 3P), spleen HSC and MPP numbers (Supplemental Figure 3Q and 3R), bone marrow cellularity, or the numbers of HSCs, MPPs, restricted hematopoietic progenitors, or myeloid, B, or T cells in the bone marrow (Figure 1E–U). Moreover, bone marrow cells from *Lyve1^Cre^; Scf^fl/fl^* mice and *Scf^fl/fl^* controls gave similar levels of multilineage reconstitution upon competitive transplantation into irradiated mice (Figure 1V). This further suggests that HSCs and progenitors in normal bone marrow do not depend upon SCF from the blood.

To test whether SCF produced in the bone marrow acts locally or systemically to promote stem and progenitor cell maintenance, we generated *Prx1^Cre^*; *Scf^fl/fl^* mice. *Prx1^Cre^* recombines in mesenchymal cells during fetal development, including in the cells that give rise to LepR^+^ bone marrow cells, in long bones (femurs and tibias) but not in vertebrae or other parts of the axial skeleton^20^. Consistent with this, *Scf* transcript levels were reduced in LepR^+^ bone marrow cells from the long bones, but not vertebrae, of *Prx1^Cre^*;*Scf^fl/fl^* as compared to *Scf^fl/fl^* controls (Supplemental Figure 4A and 4B). By ELISA analysis, *Prx1^Cre^*; *Scf^fl/fl^* mice exhibited reduced SCF levels in femur and tibia bone marrow fluid as compared to *Scf^fl/fl^* controls (approximately 40% lower; Figure 2A) but not in vertebral bone marrow fluid (Figure 2B) or blood (Figure 2C). This indicates that SCF levels in the bone marrow are primarily regulated locally.

In vertebral bone marrow, *Prx1^Cre^*; *Scf^fl/fl^* mice and *Scf^fl/fl^* controls did not significantly differ in terms of bone marrow cellularity, or the numbers of HSCs, MPPs, restricted hematopoietic progenitors, or myeloid, B, or T cells (Figure 2D–U). Furthermore, vertebral bone marrow cells from *Prx1^Cre^*; *Scf^fl/fl^* mice and *Scf^fl/fl^* littermate controls did not significantly differ in their capacity to give long-term multilineage reconstitution upon competitive transplantation into irradiated mice (Figure 3A). Conversely, in femurs and tibias, *Prx1^Cre^*; *Scf^fl/fl^* mice had significantly reduced numbers of bone marrow cells, HSCs, MPPs, and most restricted progenitors and myeloid cells as compared to *Scf^fl/fl^* controls (Figure 2D–U). Femur and tibia bone marrow cells from *Prx1^Cre^*; *Scf^fl/fl^* mice also gave significantly lower levels of myeloid, B, and T cell reconstitution upon competitive transplantation into irradiated mice (Figure 3B). Therefore, SCF produced by bone marrow mesenchymal cells regulates stem and progenitor cell maintenance by acting locally in the bone marrow compartment in which it is made.

Deletion of *Scf* from spleen and liver endothelial cells reduced SCF levels in the blood without affecting stem or progenitor cell numbers in bone marrow (Figure 1), though it is possible that Vav1-cre or Lyve1-cre also recombined in additional *Scf*-expressing cells outside the bone marrow. Either way, the observation that SCF can be depleted from the blood without affecting HSC or progenitor numbers in the bone marrow suggests that stem and progenitor cells in normal bone marrow depend mainly on SCF synthesized in the bone marrow. However, if SCF is completely eliminated from the bone marrow, SCF from the blood may at least transiently rescue HSC maintenance^16^.

Our data do not argue against the conclusion that soluble SCF is important for stem and progenitor cell maintenance^16^. Indeed, the companion study by Zhang et al. found that SCF produced within dorsal vertebrae can promote the maintenance of HSCs and progenitors in ventral vertebrae, implicating soluble SCF^25^. Nonetheless, both of our studies suggest that, under normal circumstances, SCF produced by mesenchymal stromal cells within the bone marrow promotes HSC and restricted progenitor maintenance by acting locally within the bone marrow compartment in which it is produced.

## Supplementary Material

Supplement 1

## Figures and Tables

**Figure 1.**
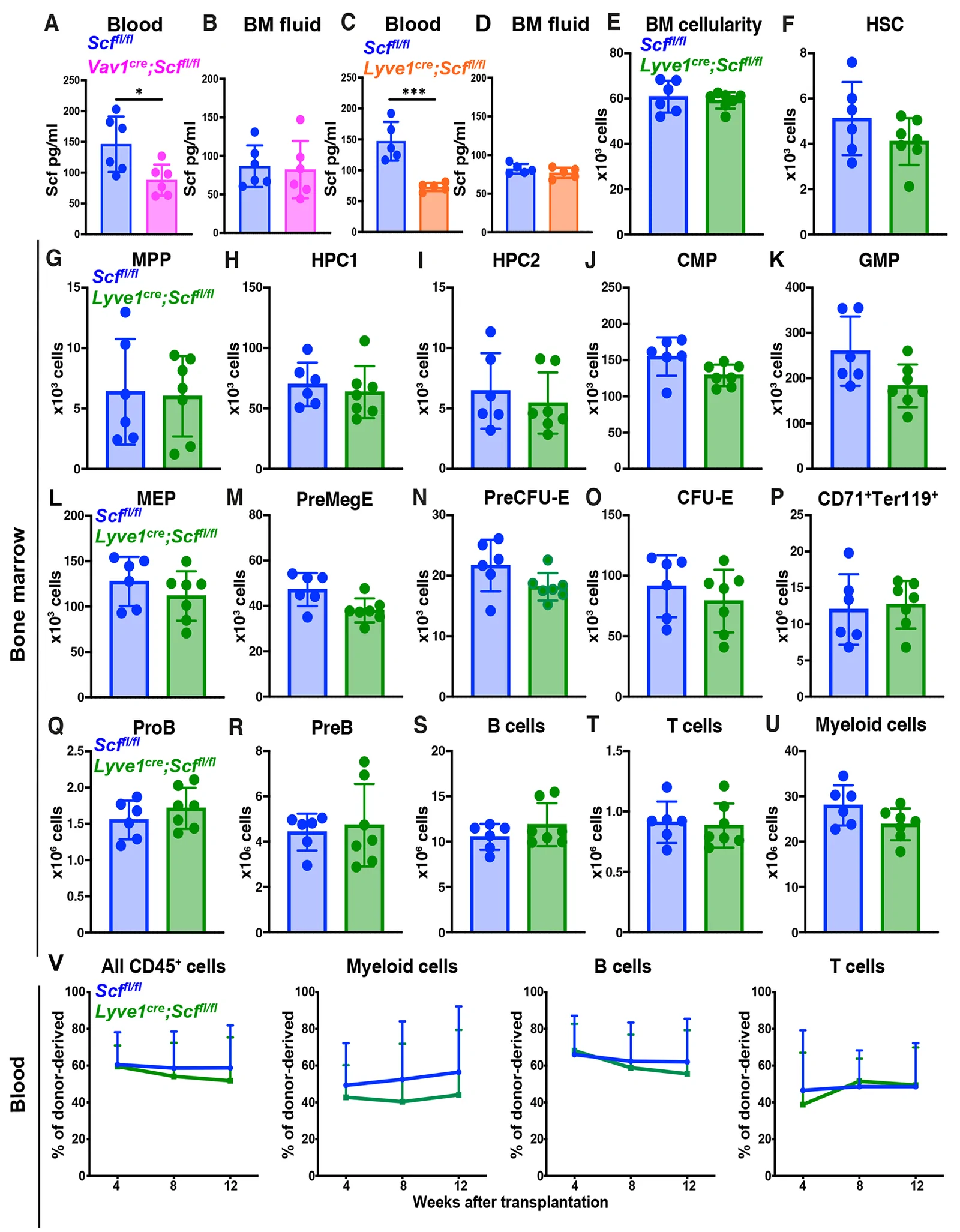
Deletion of *Scf* from spleen and liver endothelial cells reduces SCF levels in the blood without affecting the frequencies of hematopoietic stem or progenitor cells in the bone marrow. (A, B) SCF levels in blood serum (A) and bone marrow (BM) fluid collected from the femurs and tibias (B) of *Vav1*^*Cre*^*; Scf*^*fl/fl*^ and *Scf*^*fl/fl*^ control mice (a total of 6 mice per genotype from 6 independent experiments). (C, D) SCF levels in blood plasma (C) and BM fluid (D) collected from the femurs and tibias of *Lyve1*^*Cre*^*; Scf*^*fl/fl*^ and *Scf*^*fl/fl*^ control mice (a total of 5 mice per genotype from 3 independent experiments). (E-U) Bone marrow cellularity (E), as well as the numbers of HSCs (F), MPPs (G), HPC1 (H), HPC2 (I), CMPs (J), GMPs (K), MEPs (L), PreMegEs (M), PreCFU-Es (N), CFU-Es (O), CD71^+^Ter119^+^ erythroid cells (P), ProB cells (Q), PreB cells (R), B cells (S), T cells (T), and myeloid cells (U) in bone marrow from two femurs and two tibias of *Lyve1*^*Cre*^*; Scf*^*fl/fl*^ and *Scf*^*fl/fl*^ control mice (a total of 6-7 mice per genotype from 4 independent experiments). (V) Donor cell reconstitution in the blood of irradiated mice after transplantation of 500,000 donor bone marrow cells from *Lyve1*^*Cre*^*; Scf*^*fl/fl*^ or *Scf*^*fl/fl*^ control mice along with 500,000 competing wild-type cells (a total of 14-15 recipient mice per genotype transplanted with cells from 3 donors per genotype in 3 independent experiments). All data represent mean ± standard deviation. Each dot in panels A-U represents a different mouse. Statistical significance was assessed using a matched samples two-way ANOVA followed by Sidak’s multiple comparisons adjustment (A, B), Student’s *t*-tests followed by Holm-Sidak’s multiple comparisons adjustments (C, D, and F-U), a Student’s *t*-test (E), or non-parametric analysis of longitudinal data (nparLD) with the False Discovery Rate (FDR) method for multiple comparisons adjustments to test differences in overall reconstitution (V). All statistical tests were 2-sided (*, P<0.05; ***, P<0.001).

**Figure 2.**
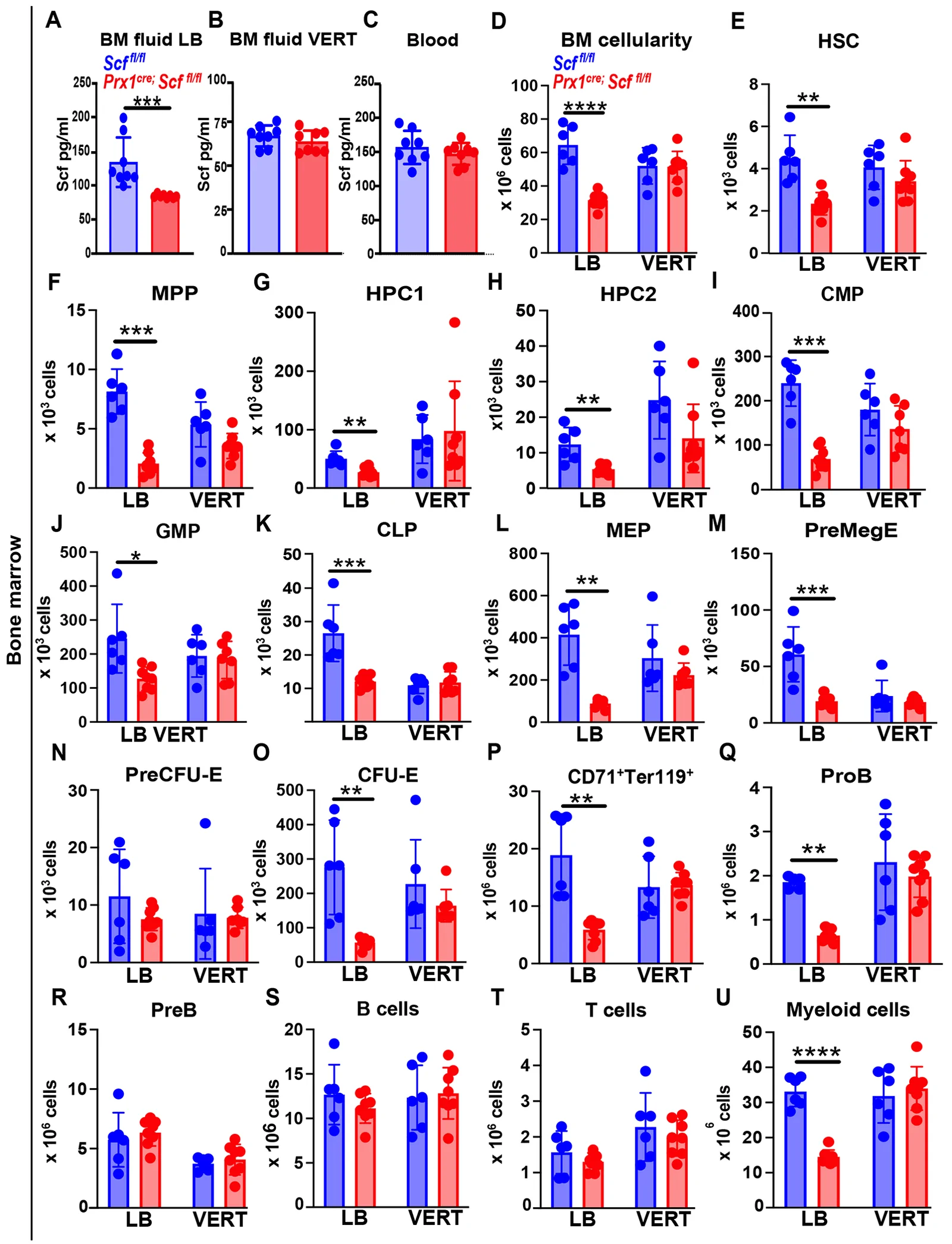
SCF acts locally within the bone marrow compartment in which it is produced to promote the maintenance of HSCs and restricted hematopoietic progenitors. *Prx1*^*Cre*^ recombines in mesenchymal cells, including LepR^+^ cells, in femurs and tibias but not in vertebrae^20^. (A-C) SCF protein levels in bone marrow fluid from the long bones (LB; femurs and tibias) (A) or vertebrae (VERT) (B), as well as from blood plasma (C) from *Prx1*^*Cre*^*; Scf*^*fl/fl*^ and *Scf*^*fl/fl*^ control mice (a total of 8 mice per genotype from 2 independent experiments). (D-U) Bone marrow cellularity (D) and the numbers of HSCs (E), MPPs (F), HPC1 (G), HPC2 (H), CMPs (I), GMPs (J), CLPs (K), MEPs (L), PreMegEs (M), PreCFU-Es (N), CFU-Es (O), CD71^+^Ter119^+^ erythroid cells (P), ProB cells (Q), PreB cells (R), B cells (S), T cells (T), and myeloid cells (U) in bone marrow from the long-bones and vertebrae of *Prx1*^*Cre*^*; Scf*^*fl/fl*^ and control *Scf*^*fl/fl*^ mice (a total of 6-8 mice per genotype from 2 independent experiments). Each dot represents a different mouse, and all data represent mean ± standard deviation. Statistical significance was assessed using Student’s *t*-tests or Mann-Whitney tests followed by Holm-Sidak’s multiple comparisons adjustments (A-C, D, and E-U). All statistical tests were 2-sided (*, P <0.05; **, P<0.01; ***, P<0.001; ****, P<0.0001).

**Figure 3.**
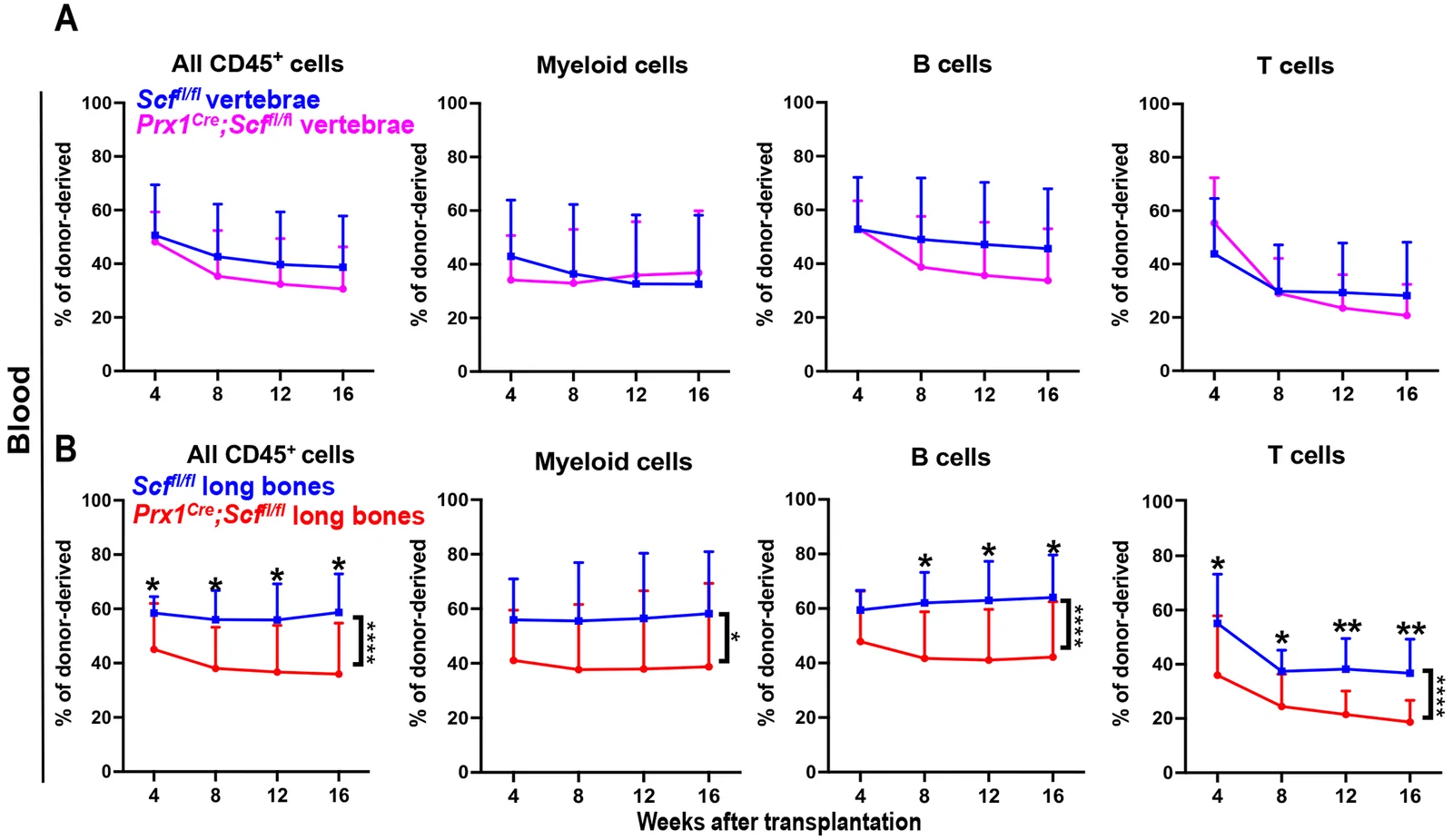
*Prx1*^*Cre*^*; Scf*^*fl/fl*^ mice have reduced reconstituting activity in bone marrow from femurs and tibias, but not vertebrae, upon competitive transplantation into irradiated mice. (A-B) Donor-derived reconstitution of CD45^+^ cells, myeloid, B, and T cells in the blood of recipient mice after competitive transplantation of 500,000 donor bone marrow cells from the vertebrae (A) or femurs and tibias (long bones; B) of *Prx1*^*Cre*^*; Scf*^*fl/fl*^ or *Scf*^*fl/fl*^ control mice, along with 500,000 competing wild-type cells, into irradiated recipients (a total of 8-10 recipient mice per genotype transplanted with cells from 2-3 donors per genotype in 2 experiments). All data represent mean ± standard deviation. Statistical significance was assessed using Student’s *t*-tests or Mann-Whitney tests followed by Holm-Sidak’s multiple comparisons adjustments for the time-point differences or nparLD tests followed by FDR multiple comparisons adjustments to test differences in overall reconstitution (A, B). All statistical tests were 2-sided (*, P <0.05; **, P<0.01; ****, P<0.0001).

## Data Availability

For original data, contact Sean.Morrison@UTSouthwestern.edu

## KEY POINTS

- *Scf* deletion in endothelial cells outside of BM reduces SCF levels in blood without affecting stem or progenitor cell frequencies in BM
- *Scf* deletion from mesenchymal cells in long bones, but not vertebrae, depletes stem and progenitor cells in long bones but not vertebrae

## Data and code availability

All data reported in this paper will be shared by the lead contact upon reasonable request.
