# Supplementary material for: Stem cell factor acts locally in the bone marrow to promote stem and progenitor cell maintenance
Source: bioRxiv. 2026 Sep 29:2026.09.28.755094. Preprint. [Version 1] doi: 10.64898/2026.09.28.755094 (PMC13648621; doi:10.64898/2026.09.28.755094)
Supplement: Supplementary file 1 [file media-1.docx]

**SUPPLEMENTAL MATERIAL**

**Supplemental Figure 1. Markers and flow cytometry gates used to identify bone marrow hematopoietic stem and progenitor cell populations.** (A) Representative flow cytometry gates used to identify hematopoietic stem and progenitor cell populations in the bone marrow, including LSK cells, LKS- myeloid progenitors, HSCs, multipotent progenitor cells (MPPs), hematopoietic progenitor cells 1 (HPC1), HPC2, common myeloid progenitors (CMPs), granulocyte-monocyte progenitors (GMPs), megakaryocyte-erythroid progenitors (MEPs), colony-forming units-erythroid (CFU-E), PreCFU-E, and PreMegE cells. (B) Representative flow cytometry gates used to identify differentiating hematopoietic cell populations in the bone marrow, including B cells, ProB cells, PreB cells, T cells, CD71^+^Ter119^+^ erythroid cells, and myeloid cells. (C) Representative flow cytometry gates used to identify common lymphoid progenitors (CLP). The HSC, MPP, HPC1, and HPC2 populations were identified as described by Oguro et al.^21^, whereas CLP, CMP, GMP, MEP, PreMegE, PreCFU-E, and CFU-E populations were identified as described by Comazzetto et al^3^ and Pronk et al^26^.

**Supplemental Figure 2. *Vav1-Cre*-mediated *Scf* deletion reduces *Scf* transcript levels in spleen, but not bone marrow, endothelial cells and does not significantly affect the multilineage reconstituting potential of bone marrow cells upon transplantation into irradiated mice.** (A, B) Relative *Scf* transcript levels in spleen (A) and bone marrow (B) endothelial cells from *Vav1^Cre^; Scf ^fl/fl^* and *Scf ^fl/fl^* control mice (a total of 3 mice per genotype from 3 independent experiments; each dot represents a different mouse). (C) Donor-derived reconstitution of CD45^+^ cells, myeloid, B, and T cells in the blood of recipient mice after competitive transplantation of 500,000 donor bone marrow cells from the femurs and tibias of *Vav1^Cre^; Scf^fl/-^* mice, *Scf^+/+^* mice, and *Scf^+/-^* mice, along with 500,000 competing wild-type cells, into irradiated recipients (5 recipient mice per genotype). Statistical significance was assessed using one-sample *t*-tests followed by Holm-Sidak multiple comparisons adjustments (A, B) or nparLD followed by FDR multiple comparisons adjustments (C). All data represent mean ± standard deviation and statistical tests were 2-sided (*, P < 0.05; **, P < 0.01; ***, P < 0.001).

**Supplemental Figure 3.** ***Lyve1-Cre* recombines in spleen and liver, but not bone marrow, endothelial cells and *Lyve1^Cre^; Scf ^fl/fl^* mice have normal steady-state hematopoiesis.** (A) The percentage of VE-cadherin⁺ endothelial cells that were tdTomato+ in bone marrow, spleen, and liver of 3-month-old *Lyve1^Cre^*; *tdTomato* mice (a total of 5–6 mice from four independent experiments). (B, C) Relative *Scf* transcript levels in spleen (B) and bone marrow (C) endothelial cells from *Lyve1^Cre^; Scf ^fl/fl^* and *Scf ^fl/fl^* control mice (a total of 3 mice per genotype from 3 independent experiments). (D-O) White blood cell (D) and red blood cell (E) counts, hemoglobin levels (F) and hematocrit (G), mean corpuscular volume (H), mean corpuscular hemoglobin (I), mean corpuscular hemoglobin concentration (J), red cell distribution width (K), and neutrophil (L), lymphocyte (M), monocyte (N) and platelet (O) counts in the blood of *Lyve1^Cre^; Scf ^fl/fl^* and littermate control (*Scf ^fl/fl^*) mice. (P-R) Spleen cellularity (P) as well as the numbers of HSCs (Q) and MPPs (R) in the spleen of *Lyve1^Cre^; Scf ^fl/fl^* and littermate control mice. Data are from a total of 6-7 mice from 4 independent experiments). All data represent mean ± standard deviation and each dot represents a different mouse. Statistical significance was assessed using a linear mixed-effects model followed by Dunnett’s multiple comparisons adjustment (A) one-sample *t*-tests followed by Holm-Sidak’s multiple comparisons adjustment (B, C) Student’s *t*-tests or Mann-Whitney tests followed by Holm-Sidak’s multiple comparisons adjustments (D-O, and Q-R) or a Student’s *t*-test (P). All statistical tests were 2-sided.

**Supplemental Figure 4. *Prx1-Cre*-mediated recombination of *Scf* reduces *Scf* transcript levels in LepR⁺ bone marrow stromal cells in long bones (femurs and tibia) but not vertebrae.** (A, B) Relative *Scf* transcript levels in LepR⁺ bone marrow stromal cells from long bones (A) versus vertebrae (B) of *Prx1^Cre^; Scf ^fl/fl^* and *Scf ^fl/fl^* control mice (a total of 3 mice per genotype from 3 independent experiments). All data represent mean ± standard deviation and each dot represents a different mouse. Statistical significance was assessed using one-sample *t*-tests followed by Holm-Sidak’s multiple comparisons adjustment (A, B).

**Supplemental Methods**

**Mice**

The *Scf ^fl^* allele (RRID:IMSR_JAX:017861) was generated in our laboratory^1^. *Lyve1^Cre^* (RRID:IMSR_JAX:012601)^18^, *Vav1^Cre^* (RRID:IMSR_JAX:008610)^19^, *Prx1^Cre^* (RRID:IMSR_JAX:005584)^20^, and *Rosa26LSL-tdTomato* reporter mice (B6.Cg-Gt(ROSA)26Sortm14(CAG-tdTomato)Hze/J; RRID:IMSR_JAX:007914) were obtained from Jackson Laboratory. All mice were maintained on a C57BL/Ka background. We used littermate or age-matched mice of both sexes that were 8-12 weeks or 4-6 months old in experiments. Mice were housed in AAALAC-accredited, specific-pathogen-free animal care facilities at UT Southwestern Medical Center (UTSW). All procedures were approved by the UTSW Institutional Animal Care and Use Committee.

**Flow cytometric analysis of hematopoietic cells and transplantation assays**

Bone marrow cells were obtained from femurs and tibias and analyzed by flow cytometry as previously described^3^. Vertebral bone marrow was obtained by removing the spinal cord and associated soft tissue, crushing vertebral bones in staining medium (Ca^2+^/Mg^2+^-free Hanks’ balanced salt solution (HBSS) supplemented with 2% heat-inactivated bovine serum), and filtering to generate single-cell suspensions. Antibodies and gating strategies for analysis of hematopoietic stem and progenitor cells, as well as mature hematopoietic cells, were as described previously^3^. Dead cells were excluded by propidium iodide staining. Cells were analyzed using a BD FACSLyric cytometer. For competitive transplantation, 500,000 unfractionated bone marrow cells from donor (CD45.2) and competitor (CD45.1) mice were mixed and injected into the retro-orbital venous sinus of irradiated (CD45.1/CD45.2) recipients. Peripheral blood chimerism was assessed every 4 weeks by flow cytometry as previously described^3,24,25^. HSC, MPP, HPC1, and HPC2 populations were identified as previously described by Oguro et al.^21^, whereas CLP, CMP, GMP, MEP, PreMegE, PreCFU-E, and CFU-E populations were identified as described by Comazzetto et al^3^ and Pronk et al^26^. Markers and flow cytometry gates used to identify bone marrow hematopoietic stem and progenitor cell populations are shown in Supplemental Table 1 and Supplemental Figure 1.

**ELISA analysis**

Blood was collected into ethylenediaminetetraacetic acid (EDTA)-containing tubes (to prevent clotting) to obtain plasma or empty tubes to obtain serum. The samples were centrifuged at 2,400 x g for 5 minutes to separate plasma or serum from the cellular fraction or clot, respectively. SCF concentrations were measured in the plasma or serum using the Mouse SCF Quantikine ELISA Kit (R&D Systems, MCK00) according to the manufacturer’s instructions. SCF levels were measured in blood serum, blood plasma, or bone marrow fluid from long bones (femurs and tibias) or vertebrae. Bone marrow fluid was obtained from long bones by cutting the ends off femurs and tibias, then centrifuging the bones at 2,400 x g for 5 minutes to centrifuge the bone marrow out of the bones and into 10 ul of phosphate-buffered saline (PBS), then the supernatant was collected. Vertebral bone marrow fluid was prepared by crushing vertebrae in PBS, followed by centrifugation to collect the supernatant.

**Isolation of endothelial cells and LepR^+^ stromal cells and RT-qPCR analysis**

Bone marrow and spleen endothelial cells and bone marrow LepR⁺ stromal cells were isolated as previously described^2,9^. Bone marrow cells were obtained from crushed femurs and tibias and enzymatically dissociated in HBSS containing DNase I (200 U/mL), Dispase (4 mg/mL), and collagenase I (3 mg/mL) for 30 minutes at 37°C. Spleens were mechanically dissociated and enzymatically digested as previously described^2,9^. Cells were stained with biotinylated anti-LepR (AF497; R&D Systems), anti-CD45 (30-F11; APC-eFluor 780; eBioscience), anti-Ter119 (TER-119; APC-eFluor 780; eBioscience), anti-CD31 (clone 390; PE/Cyanine7-conjugated, BioLegend, 102418), and streptavidin (BV421-conjugated, 405226; BioLegend). Dead cells were excluded using Ghost Dye Red 780 (13-0865-T100; Tonbo Biosciences). Cells were sorted using a FACSAria II (BD Biosciences) directly into TRIzol. Total RNA was extracted according to the manufacturer’s instructions, and cDNA was synthesized using the iScript cDNA Synthesis Kit (Bio-Rad). Quantitative real-time PCR was performed using iTaq Universal SYBR Green Supermix (Bio-Rad). *Scf* expression was normalized to *Actb* (which encodes β-actin), and relative expression was calculated using the 2^⁻ΔΔCt^ method. Primers were as previously described by Ding et al.¹: *Scf,* forward 5′-TTGTTACCTTCGCACAGTGG-3′ and reverse 5′-AATTCAGTGCAGGGTTCACA-3′; *Actb*, forward 5′-GCTCTTTTCCAGCCTTCCTT-3′ and reverse 5′-CTTCTGCATCCTGTCAGCAA-3′.

**Statistical methods**

In each type of experiment, multiple mice were tested in multiple independent experiments performed on different days. Mice were allocated to experiments randomly and samples processed in an arbitrary order, but formal randomization techniques were not used. No formal blinding was applied when performing the experiments or analyzing the data. Sample sizes were not pre-determined based on statistical power calculations but were based on our experience with these assays. No data were excluded. Prior to analyzing the statistical significance of differences among treatments, we tested whether data were normally distributed and whether variance was similar among groups.

To test for normality, we performed Shapiro–Wilk tests when 3≤n<20 or D’Agostino-Pearson omnibus normality tests when n≥20. To test whether variability significantly differed among groups we performed F-tests (for experiments with two groups) or Levene’s median tests (for experiments with more than two groups). When the data significantly deviated from normality or variability significantly differed among groups, we log2-transformed the data and tested again for normality and variability. If the transformed data no longer significantly deviated from normality and equal variability, we performed parametric tests on the transformed data. If log2-transformation was not possible or the transformed data still significantly deviated from normality or equal variability, we performed non-parametric tests on the non-transformed data.

When data or log2-transformed data were normal and equally variable, statistical analysis was performed using Student’s *t*-tests (when there were two groups), matched samples two-way ANOVAs (when there were two or more groups), or mixed effects analyses (when there were missing values but the data otherwise met the assumptions for matched samples ANOVAs). When the data and log2-transformed data were abnormal or unequally variable, statistical analysis was performed using Mann-Whitney tests (when there were two groups), Kruskal-Wallis tests (when there were more than two groups), or nparLD tests (when there were two or more groups measured at multiple time points). P-values from multiple comparisons were adjusted using Tukey’s (for all possible comparisons), Sidak’s (for planned comparisons), or Dunnett’s methods (for comparisons between the control and treatment groups) following ANOVAs or mixed effects analyses, Dunn’s method following Kruskal-Wallis tests, or Benjamini-Hochberg’s False Discovery Rate (FDR) method following nparLD tests. Holm-Sidak’s method was used to adjust comparisons following *t*-tests or Mann-Whitney tests. All statistical tests were two-sided where applicable. All data represent mean ± standard deviation. Statistical tests were performed using GraphPad Prism V11.0.1 or R 4.5.0.

**Supplemental References**

26. Pronk CJ, Rossi DJ, Mansson R, et al. Elucidation of the phenotypic, functional, and molecular topography of a myeloerythroid progenitor cell hierarchy. *Cell Stem Cell*. 2007;1(4):428–442.

**Supplemental Table 1.** Antibodies and viability reagents used for flow cytometry.

| **ANTIBODY OR REAGENT** | **FLUOROCHROME** | **CATALOG NUMBER** |
| --- | --- | --- |
| CD150 (TC15-12F12.2; BioLegend) | BV421 | 115925 |
| CD2 (RM2-5; Tonbo Biosciences) | FITC | 35-0021 |
| CD3 (17A2; BioLegend) | FITC | 100203 |
| CD5 (53-7.3; BioLegend) | FITC | 100605 |
| CD8α (53-6.7; Tonbo Biosciences) | FITC | 35-0081 |
| B220/CD45R (RA3-6B2; Tonbo Biosciences) | FITC | 35-0452 |
| Ter119 (TER-119; Tonbo Biosciences) | FITC | 35-5921 |
| Gr-1/Ly-6G/Ly-6C (RB6-8C5; Tonbo Biosciences) | FITC | 35-5931 |
| CD127/IL-7Rα (A7R34; Tonbo Biosciences) | PE | 50-1271 |
| Viability reagent (BD Pharmingen) | Propidium iodide (PI) | 556463 |
| Sca-1/Ly-6A/E (D7; Thermo Fisher Scientific/eBioscience) | PE-Cyanine7 | 25-5981-82 |
| CD48 (HM48-1; BioLegend) | Alexa Fluor 700 | 103425 |
| CD135/Flt3 (A2F10; BioLegend) | APC | 135310 |
| c-Kit/CD117 (2B8; Thermo Fisher Scientific/eBioscience) | APC-eFluor 780 | 47-1171-82 |
| CD105/Endoglin (MJ7/18; BD Biosciences) | BV421 | 562760 |
| CD34 (RAM34; Thermo Fisher Scientific/eBioscience) | FITC | 11-0341-82 |
| CD16/32/FcγRIII/II (93; BioLegend) | BV510 | 101333 |
| CD2 (RM2-5; Tonbo Biosciences) | PE | 50-0021 |
| CD3 (17A2; Tonbo Biosciences) | PE | 50-0032 |
| CD5 (53-7.3; BioLegend) | PE | 100607 |
| CD8α (53-6.7; Tonbo Biosciences) | PE | 50-0081 |
| B220/CD45R (RA3-6B2; Tonbo Biosciences) | PE | 50-0452 |
| Ter119 (TER-119; Tonbo Biosciences) | PE | 50-5921 |
| Gr-1/Ly-6G/Ly-6C (RB6-8C5; Tonbo Biosciences) | PE | 50-5931 |
| CD41 (MWReg30; BioLegend) | Alexa Fluor 700 | 133926 |
| CD150 (TC15-12F12.2; BioLegend) | APC | 115909 |
| CD71/Transferrin receptor (RI7217; BioLegend) | BV421 | 113813 |
| Gr-1/Ly-6G/Ly-6C (RB6-8C5; BioLegend) | BV510 | 108437 |
| CD43 (S7; BD Biosciences) | PE | 553271 |
| Ter119 (TER-119; BioLegend) | PE-Cyanine7 | 116222 |
| CD3 (17A2; Thermo Fisher Scientific/eBioscience) | Alexa Fluor 700 | 56-0032-82 |
| IgM (II/41; Thermo Fisher Scientific/eBioscience) | APC | 17-5790-82 |
| Mac-1/CD11b (M1/70; Thermo Fisher Scientific/eBioscience) | APC-eFluor 780 | 47-0112-82 |
| LepR (polyclonal; R&D Systems; biotinylated) | Biotin | AF497 |
| Streptavidin (BioLegend) | BV421 | 405226 |
| CD45 (30-F11; Thermo Fisher Scientific/eBioscience) | APC-eFluor 780 | 47-0451-82 |
| Ter119 (TER-119; Thermo Fisher Scientific/eBioscience) | APC-eFluor 780 | 47-5921-82 |
| Ghost Dye Red 780 viability dye (Tonbo Biosciences) | Ghost Dye Red 780 | 13-0865-T100 |
| CD31 (390; BioLegend) | PE/Cyanine7 | 102418 |
